# The diagnostic and therapeutic challenges of pyoderma gangrenosum in a 32-year-old woman with systemic lupus erythematosus: a case report

**DOI:** 10.1097/MS9.0000000000004119

**Published:** 2025-10-22

**Authors:** Anjuman Ara Rahman, Abhijit Datta, Sabiha Sal Sabil, Anindita Das Barshan, Mohammad Jahid Hasan

**Affiliations:** a Public Health, North South University, Dhaka, Bangladesh; b Rheumatology, Sher-E-Bangla Medical College Hospital, Barishal, Bangladesh; c Radiotherapy & Oncology, Dhaka Medical College Hospital, Dhaka, Bangladesh; d Tropical Disease, Tropical Disease and Health Research Center, Dhaka, Bangladesh; e Public Health, Pi Research & Development Center, Dhaka, Bangladesh

**Keywords:** biological therapy, etanercept, immunosuppressive agents, lupus erythematosus, pyoderma gangrenosum, systemic

## Abstract

**Introduction and importance:**

The occurrence of (PG) in the context of systemic lupus erythematosus (SLE) can be challenging to diagnose and might complicate disease management and affect patient outcomes. This case report aims to add evidence on PG occurring during inactive SLE and highlights the role of tumor necrosis factor alpha inhibition in refractory cases where conventional therapy failed.

**Case presentation:**

A 32-year-old woman with an 8-year history of SLE presented with multiple painful, necrotic ulcers over both lower limbs. Diagnosis was based on clinical presentation, exclusion of infections, and vasculitis. Significant improvement was observed following the initial treatment with high-dose prednisolone (50 mg daily). Nevertheless, reducing the dosage to 7.5 mg per day exacerbated the lesion. Following an unsatisfactory response by prednisolone, azathioprine at a daily dose of 150 mg, and mycophenolate mofetil at a daily dose of 2 g, etanercept (25 mg every other week) was administered. Administration of 25 mg of etanercept for a period of 3 months resulted in satisfactory healing of the ulcers.

**Clinical discussion:**

This case highlights the complexities of diagnosing and managing PG in the context of SLE, particularly in patients with inactive lupus. The treatment journey underscores the importance of individualized therapeutic strategies, including the potential use of biologic agents like etanercept in refractory cases. It also sheds light on maintenance of initial corticosteroid therapy and reconsideration of early dose tapering.

**Conclusion:**

Some uncommon and challenging presentation of pyoderma gangrenosum in a SLE patient may require an appropriate, individualized management.

## Introduction

Pyoderma gangrenosum (PG) is a rare, neutrophilic dermatosis characterized by pustules and necrotic ulcers with undermined erythematous borders^[1]^. PG presents a diagnostic and therapeutic challenge due to the limited understanding of its complex pathogenesis. The etiology of PG remains largely idiopathic and characterized by abnormal accumulation of neutrophil and immune mediators in the skin without any infectious agent^[2]^. Furthermore, it frequently coexists with systemic diseases, most notably inflammatory bowel disease (IBD), rheumatoid arthritis (RA), systemic lupus erythematosus (SLE), and hematological disorders^[3]^. The occurrence of PG in patients with SLE is uncommon. Several case reports have been published on PG in individuals with both active and inactive SLE, each emphasizing the difficulties in diagnosing and treating PG in this particular situation^[4,5]^. We present a case report of a 32-year-old woman with SLE with hypothyroidism who developed PG with inactive SLE disease activity. However, it is important to understand this atypical association because it needs an early diagnosis and tailored treatment plans. This case report aims to provide valuable insights into managing PG in the context of SLE by sharing the diagnostic challenges, treatment options, and patient outcomes, ultimately raising awareness among healthcare providers and improving diagnosis and treatment in such complex cases. Furthermore, it may offer additional evidence that PG can arise during inactive SLE and to emphasize the importance of TNF-α suppression in refractory cases when traditional therapy has failed.

**HIGHLIGHTS**

- The uncommon association of pyoderma gangrenosum with inactive systemic lupus erythematosus can complicate the difficulty of diagnosis and treatment in such cases.
- Despite initial improvement, sudden worsening of ulcers can occur during corticosteroids tapering.
- This case highlights the significance of personalized treatment approaches, especially early initiation of biologic agents such as etanercept for refractory cases of pyoderma gangrenosum with systemic lupus erythematosus.

## Case report

A 32-year-old female hailing from Faridpur with a well-documented history of SLE with hypothyroidism for 8 years, presented with multiple, painful, rounded ulcers on the anterior aspect of lower limbs for 4 months. These lesions began as small, erythematous papules on the anterior tibial areas and rapidly progressed into large necrotic ulcers within a few days. The patient reported significant pain disproportionate to the size of the ulcers and noted purulent discharge from the lesions. She denied any recent joint pain, malar rash, anuria or oliguria, edema, bleeding manifestation, trauma, infections, or new medications. Her SLE, primarily involving the skin and joints, was in remission with hydroxychloroquine 300 mg/day and there was no recent exacerbation. She was in euthyroid status managed with levothyroxine 125 µg per day. Before development of PG, she was apparently stable with her current medication regimen for the past 6 months. She received multiple antibiotic treatments prior to coming to our center, but there was no improvement.

Physical examination revealed multiple well-demarcated, irregularly shaped ulcers on both lower limbs, with the largest measuring approximately 2.5 cm in diameter on the right leg. The ulcers had a purulent and necrotic base, as well as erythematous undermined edges. The largest one extended up to the superficial fascia. The surrounding skin showed signs of inflammation but no significant induration or lymphadenopathy. The patient also exhibited signs of mild systemic involvement, including low-grade fever and fatigue.

In order to establish the diagnosis of PG and eliminate other possible causes of ulcerative lesions, a thorough diagnostic evaluation was performed based on the patient’s clinical presentation. Complete blood count showed mild leukocytosis with a white blood cell count of 12 000/µL and mild anemia consistent with chronic disease (hemoglobin 9.5 g/dL). Erythrocyte sedimentation rate and C-reactive protein were found to be 6 and 11 mg/L, respectively, supporting the presence of inflammation.

Her autoimmune investigations revealed positive anti-ds-DNA (anti-double-stranded DNA) antibody with low titer, low positive ACLA (anti-cardiolipin antibody) IgG (immunoglobulin G) and IgM (immunoglobulin M), negative anti-B-2 Glycoprotein-1 antibodies (IgG and IgM), negative lupus anticoagulant antibody, and negative anti phospholipid antibodies (IgG and IgM). Biopsy was not performed due to financial issue. It was a clinical diagnosis, ruling out infectious causes. Other investigations such as renal and liver function tests and serum thyroid stimulating hormone test were normal.

## Diagnosis

Based on the clinical presentation and laboratory findings, a diagnosis of PG associated with inactive SLE and hypothyroidism was established. The diagnostic criteria for ulcerative type PG was proposed by the Delphi consensus exercise using the RAND/ University of California at Los Angeles (UCLA) Appropriateness Method^[6]^. The updated diagnostic criteria for PG have a sensitivity of 86% and a specificity of 90%^[7]^. To diagnose PG, one major criterion and four out of eight minor criteria must be met. The major criterion is a biopsy of the ulcer edge showing a neutrophilic infiltrate. The eight minor criteria are as follows: (a) exclusion of infection; (b) pathergy; (c) history of inflammatory bowel diseases or inflammatory arthritides; (d) history of papule, pustule, or vesicle ulcerating within 4 days of appearing; (e) peripheral erythema, undermining border, and tenderness at the ulceration site; (f) multiple ulcerations, with at least one on an anterior lower leg ulcer site; (g) cribriform or “wrinkled paper” scars at healed ulcer sites; and (h) decreased ulcer size within 1 month of starting immunosuppressive therapy^[7]^. Unfortunately, to follow the diagnostic criteria, we suggested a biopsy from the lesion, which the patient could not afford due to an already high out-of-pocket expense of her treatment. Our diagnosis was confirmed based on the clinical features and fulfilling four minor criteria (exclusion of infection, history of papule, undermining border, and multiple ulcerations on an anterior lower leg ulcer site).

## Treatment

**Figure 1.**
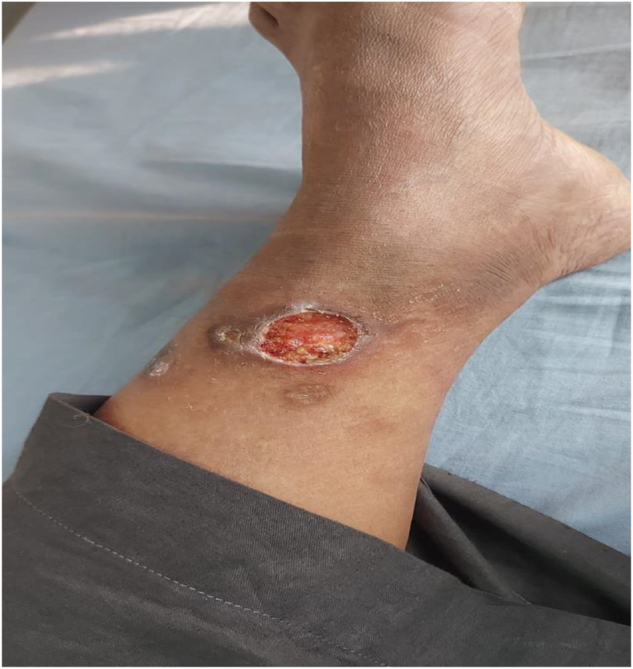
Active pyoderma gangrenosum ulcers with undermined erythematous borders and necrotic bases on the anterior lower leg before biologic treatment. Largest ulcer on right anterior tibia: ~ 2.5 cm diameter, estimated area ~ 4.9 cm^2^. Ulcers had purulent discharge and undermined edges.

**Figure 2.**
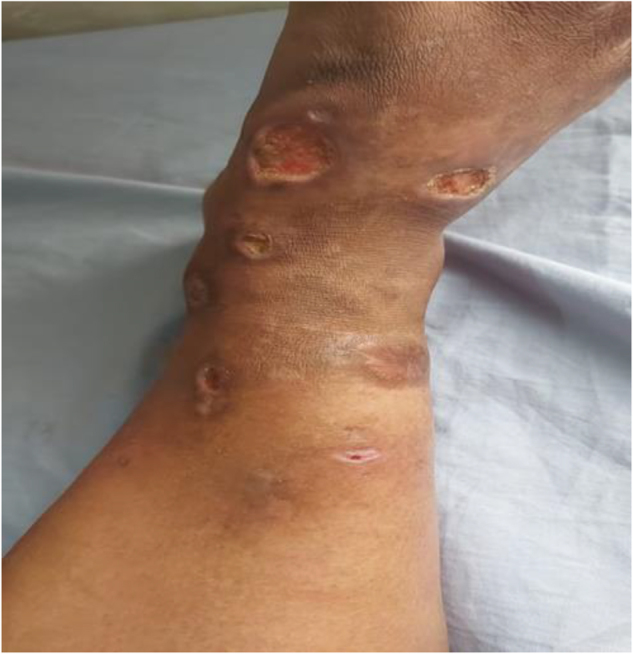
After 3 months of mycophenolate mofetil (2 g/day): Minimal change. Larger circular ulcers (largest ulcer ~ 2.0 cm, area ~ 4.1 cm^2^) with granulating base and inflamed margins at presentation, prior to etanercept therapy.

**Figure 3.**
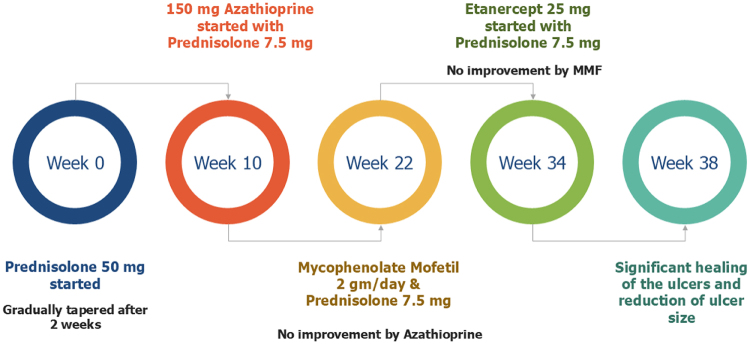
Treatment timeline: poor response to mycophenolate, rapid ulcer healing with etanercept, and complete sustained remission by 3 months.

The patient was prescribed 50 mg of prednisolone daily. The response to prednisolone was dramatic, initially reducing the purulent discharge and healing of the base. After 2 weeks, a gradual tapering of the dosage of prednisolone was initiated as improvements were consistent (Figs. 1, 2). However, the lesions started to deteriorate at 10 weeks when the prednisolone dosage was reduced to 7.5 mg daily. Consequently, a daily regimen of 150 mg azathioprine was introduced with a low-dose steroid. Unfortunately, there was no significant improvement after 3 months of azathioprine treatment; therefore, Mycophenolate mofetil (MMF) , 2 gm/day, was prescribed. After another 3 months of MMF, the response remained unsatisfactory. Treatment was then shifted to etanercept, administered at 25 mg every other week. The response to etanercept was satisfactory, with significant healing of the ulcers and reduction of ulcer size within 1 month. Prednisolone was gradually discontinued over the next 2 months (Fig. 3).

## Outcome of patient

**Figure 4.**
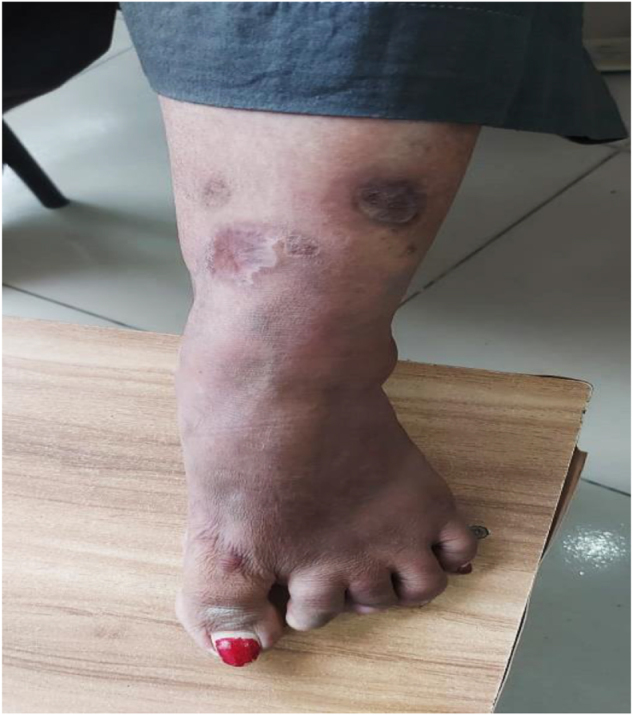
Healed ulcers (largest ulcer ~ 1.0 cm, area ~ 0.8 cm^2^, total ulcer area reduced >70%) with complete epithelialization and residual post-inflammatory hyperpigmentation on the anterior lower leg at 1 month.

**Figure 5.**
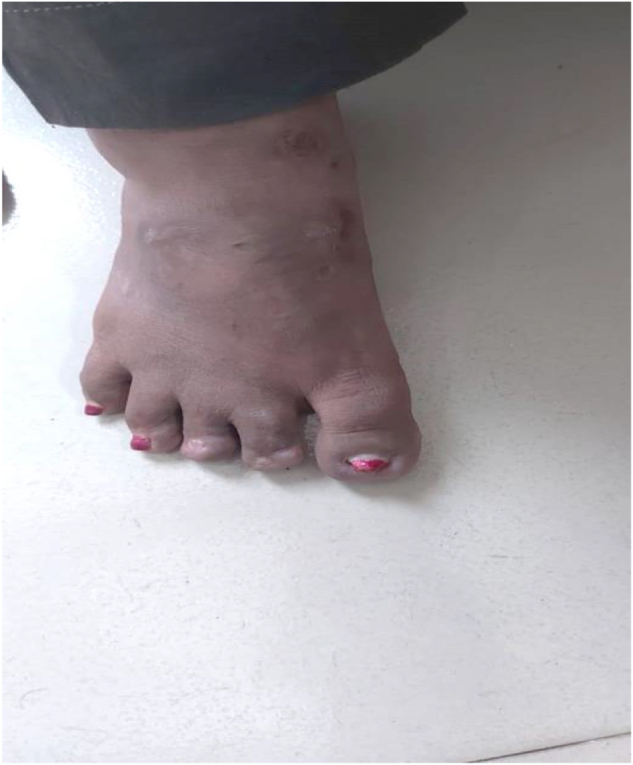
Healed ulcers with complete epithelialization and residual post-inflammatory hyperpigmentation on the anterior lower leg at 3-month follow-up after etanercept therapy.

The largest ulcer was on the right anterior tibia, which measured approximately 2.5 cm in diameter and had an estimated area of 4.9 cm^2^, exhibiting purulent discharge and undermined margins at the baseline. The largest ulcer was reduced to only 2.0 cm in diameter (area 4.1 cm^2^) after 3 months of mycophenolate mofetil (2 g/day) with no significant change in edges. However, this same ulcer was reduced to 1.0 cm (area 1.8 cm^2^) and the total ulcer area was reduced by over 70% after 1 month of etanercept (25 mg every other week). Consequently, significant healing was observed. Complete epithelialization of all ulcers was attained after three months of etanercept treatment, and the total ulcer area was 0 cm^2^. No recurrence was observed during the 6-month follow-up period. (Figs. 4, 5).

## Discussion

PG presents a significant challenge, particularly when occurring in conjunction with SLE, as highlighted by this case involving a 32-year-old woman. PG most commonly affects adults between the ages of 20 and 40, with a no sex predominance^[8]^. In a previous literature review, the mean age of patients suffering from PG with SLE was reported as 39.2 (15.9)^[9]^. This case report of a 32-year-old woman fits within the typical age range for PG, underscoring the relevance of age as a contributing factor in the presentation and management of this condition.

Our patient presented with classical type of PG, which is the commonest variety occurring in 85% cases^[10]^. The classic form of PG is characterized by the rapid onset of painful ulcers with raised, undermined borders. It is also known as systemic PG, which is frequently associated with underlying systemic diseases such as IBD, RA and, less commonly, SLE. Another form, bullous PG, presents as fluid-filled blisters that quickly rupture, leading to necrotic base ulcers. Pustular PG is identified by the formation of multiple small pustules that coalesce into painful ulcers. Vegetative PG is marked by the development of skin lesions that appear as cauliflower-like growths, posing significant management challenges. Additionally, there is postoperative PG, which typically arises following surgical procedures and is associated with trauma or skin injury^[10]^.

This patient presented with the ulcers in the anterior aspects of lower limb which is the commonest area of PG^[11]^. There are several case reports which reported to have a high or moderately active lupus flare along with the occurrence of PG^[4,12]^. In such scenarios, the immunological dysregulation inherent in SLE, including aberrant neutrophil activity and immune complex deposition, can provoke inflammatory responses in the skin, leading to PG development. However, this patient developed PG with remission and stable phase of SLE. This event warrants further understanding about the independent development of PG irrespective of disease activity level. A few case reports have documented development of PG during periods of clinical remission in SLE patients, indicating that underlying subclinical immune dysregulation may persist and contribute to PG even when lupus symptoms are not active^[13]^.

Diagnosing PG in patients with SLE is complicated owing to the overlapping clinical features of both conditions. There are multiple diagnostic criteria present to diagnose PG, although it mostly remains a clinical diagnosis of exclusion. In the context of diagnosing PG in SLE, biopsy can be a crucial tool for differentiating PG from other ulcerative or inflammatory skin conditions. Unfortunately, in our case, a biopsy could not be performed. Despite the absence of biopsy results, the clinical presentation, absence of infectious agent, and vasculitis along with the patient’s medical history and laboratory findings, supported a clinical diagnosis of PG. Although biopsy is an important diagnostic tool and a major criterion for PG, it could not be performed in this case due to significant financial constraints. The diagnosis was based on validated Delphi minor criteria—exclusion of infection and characteristic clinical features. A comprehensive understanding of the clinical symptoms is of great help in case synthesis and in creating a useful contribution to patient management, since the diagnosis of PG is not dependent just on the biopsy results in isolation from other studies^[14]^.

The management of PG in SLE patients often involves complex, trial-and-error strategies due to variable treatment responses. Our patient responded well with the initial therapy with high-dose prednisolone but tapering the dosage led to deterioration of lesion. Subsequent treatments with azathioprine and MMF were unsuccessful, necessitating a switch to etanercept, which ultimately led to complete resolution. Interleukin-1(IL-1) and TNF-α cytokines play a role in the immunopathogenesis of neutrophilic dermatosis, supporting their classification as autoinflammatory diseases. As a result, TNF-α inhibitors (e.g., infliximab, adalimumab, etanercept, certolizumab pegol, and golimumab) and IL-1 inhibitors (e.g., anakinra) are becoming the preferred treatment for PG, although glucocorticoids remain the mainstay^[15]^. A randomized control trial by Brooklyn et al. found that infliximab (IFX) at a dose of 5 mg/kg was more effective than placebo in treating PG (response rate 69%), while 31% did not respond to IFX^[16]^. However, a semi-systematic review by Abdallah et al. discovered no significant difference in the effectiveness of 3 TNF-α inhibitors-IFX, adalimumab, and etanercept^[17]^. On the contrary, in another study, etanercept was found both safe and efficacious when used to treat refractory PG. The safety and effectiveness of etanercept in treating PG following IFX serum sickness were also reported in another case report previously^[18]^. Regarding IL-1 inhibitors, it has been observed that 1-10% of individuals receiving anakinra treatment are predicted to experience a serious infection^[19]^. These evidences underscore the necessity of picking etanercept instead of IFX and anakinra in our patient. Recent research has shown that spesolimab, an anti-interleukin-36 R antibody, may be used to treat neutrophil-driven dermatoses like refractory PG, especially in individuals who are not responding to TNF-α inhibitors^[20]^. However, spesolimab is not currently available in Bangladesh. This challenging treatment plan in our case emphasizes the need for flexibility and patience in treatment approaches and the potential requirement for advanced immunosuppressive therapies when conventional treatments fail. Although this experience is discordant with some previous case reports, where only glucocorticoid successfully reversed the condition,^[4,5,12]^ some earlier case studies also documented MMF as an effective immunosuppressive for refractory PG^[21]^. All of these studies highlight the importance of tailored treatment plans based on improvement of the lesions and the early initiation of immunosuppressives, where the steroid fails to respond, and biologics in the management of complicated PG cases associated with SLE.

## Limitation

This report describes a treatment journey of a single patient, which limits the generalizability of the findings. A biopsy is a major diagnostic criterion for diagnosing pyoderma gangrenosum which could not be performed in this case due to both financial constraints and patient’s reluctance. Thus, the diagnosis relied on validated minor clinical criteria and exclusion of other causes. Histopathology would have strengthened the case, and we recognize this as a limitation and it reflects real-world practice in resource-limited settings. Additionally, quantitative wound measurements were limited to clinical observation and photographic assessment rather than standardized digital planimetry. Further studies are required to find out the specific treatment options in case of refractory PG with inactive SLE.

## Conclusion

This case report sheds light on the challenges involved in diagnosing and managing PG in the context of SLE. The occurrence of PG in our 32-year-old patient, despite inactive lupus, underscores the separate immune reaction for the presentation of PG independently. Our limitation includes the inability to perform a biopsy to confirm the diagnosis which led to a diagnosis of exclusion based on this clinical context. The treatment approaches involving multiple biologics before achieving resolution with etanercept reflects the therapeutic dilemmas often encountered in such cases. This case will reinforce the importance of patient-specific treatment strategies and will serve as evidence on the effective use of biologic agents for refractory PG in SLE patients.

## Data Availability

All data were available to the lead author and could be found upon the reasonable request to the corresponding author.
